# 

**Published:** 1897-11

**Authors:** 


					﻿ESTABLISHED IS55.
SUBSCRIPTION, S2.0S.
ATLANTA
JIEDICflli MD SUR6MI1
JOURNAL. w-3~'wnJ
newIefjes. Atlanta, Ga., November, 1897. No. 9.
				

